# Rare bilateral anatomical variation of the lateral thoracic artery: duplicated arteries with unique origins and pathways

**DOI:** 10.1007/s00276-025-03587-y

**Published:** 2025-02-18

**Authors:** Mark Rimmer, Warwick J. Peacock, Kalyanam Shivkumar, Shumpei Mori

**Affiliations:** 1 Center for Interventional Programs, UCLA Health System, and the UCLA Cardiac Arrhythmia Center & EP Programs, David Geffen School of Medicine, Los Angeles, CA USA; 2 Department of Surgery, UCLA, Los Angeles, CA USA

**Keywords:** Anatomy, Lateral thoracic artery, Variation

## Abstract

The lateral thoracic artery (LTA) is one of six main branches that originate from the axillary artery. The LTA has a textbook origin from the 2nd part of the axillary artery posterior to the pectoralis minor muscle. Contrary to the textbook origin, there are numerous reports of LTA variants that originate from the thoracoacromial artery, subscapular artery, and thoracodorsal artery, or even its duplication. This case report involves description of an additional unique duplicate variant of the LTA, bilaterally. The right duplicate LTA has its origin from the 3rd part of the axillary artery and then courses with the axillary sheath before coursing towards the breast. The right duplicate LTA variant also gives off two small subcutaneous branches in the medial upper arm. The left duplicate LTA has its origin from the brachial artery and courses directly to the breast through the axilla. Additional detail on the variants of the LTA could prove useful in surgical procedures that involve the lateral thorax, chest, and axilla and contribute to broader anatomical knowledge.

**Supplementary Information:**

The online version contains supplementary material available at 10.1007/s00276-025-03587-y.

## Introduction

The axillary artery is divided into three parts in relation to the pectoralis minor muscle. The 1st, suprapectoral, part courses from the lateral border of the 1st rib to the superior border of the pectoralis minor. The 2nd, retropectoral, part lies posterior to pectoralis minor. The 3rd, infrapectoral, part continues from the inferior border of pectoralis minor to the inferior border of the teres major muscle. The lateral thoracic artery (LTA) usually originates from the 2nd part distal to the thoracoacromial artery (Supplementary Figure) according to classic anatomical descriptions and text. It courses along the lateral chest wall with the lateral thoracic vein (LTV) deep to the pectoralis minor, emerging inferior to the muscle belly, and it terminates by giving off lateral mammary, muscular, and cutaneous branches [2, 9].

The LTA supplies blood to tissues of the lateral thorax. Primary recipient tissues include the intermediate portion of the serratus anterior muscle, intercostal muscles, pectoral muscles, axillary lymph nodes, and lateral chest wall and skin. In females, the LTA has a greater role as it perfuses the lateral breast [2, 9]. Furthermore, in females, the LTA also serves as an important vessel contributing to the major blood supply of the nipple-areolar complex [5].

The LTA has been reported to vary in its origin from the axillary artery [2]. We herein demonstrate a rare case of bilateral duplicated LTA variance. Two LTAs were found bilaterally with both 2nd LTAs demonstrating unique origins and pathways. Understanding of potential and existing variations is practically important for surgeons that operate in the vicinity of the LTA, including procedures involving the breast, pectoral muscles, or broader lateral thorax, chest, and axilla.

## Case report

**Fig. 1:**
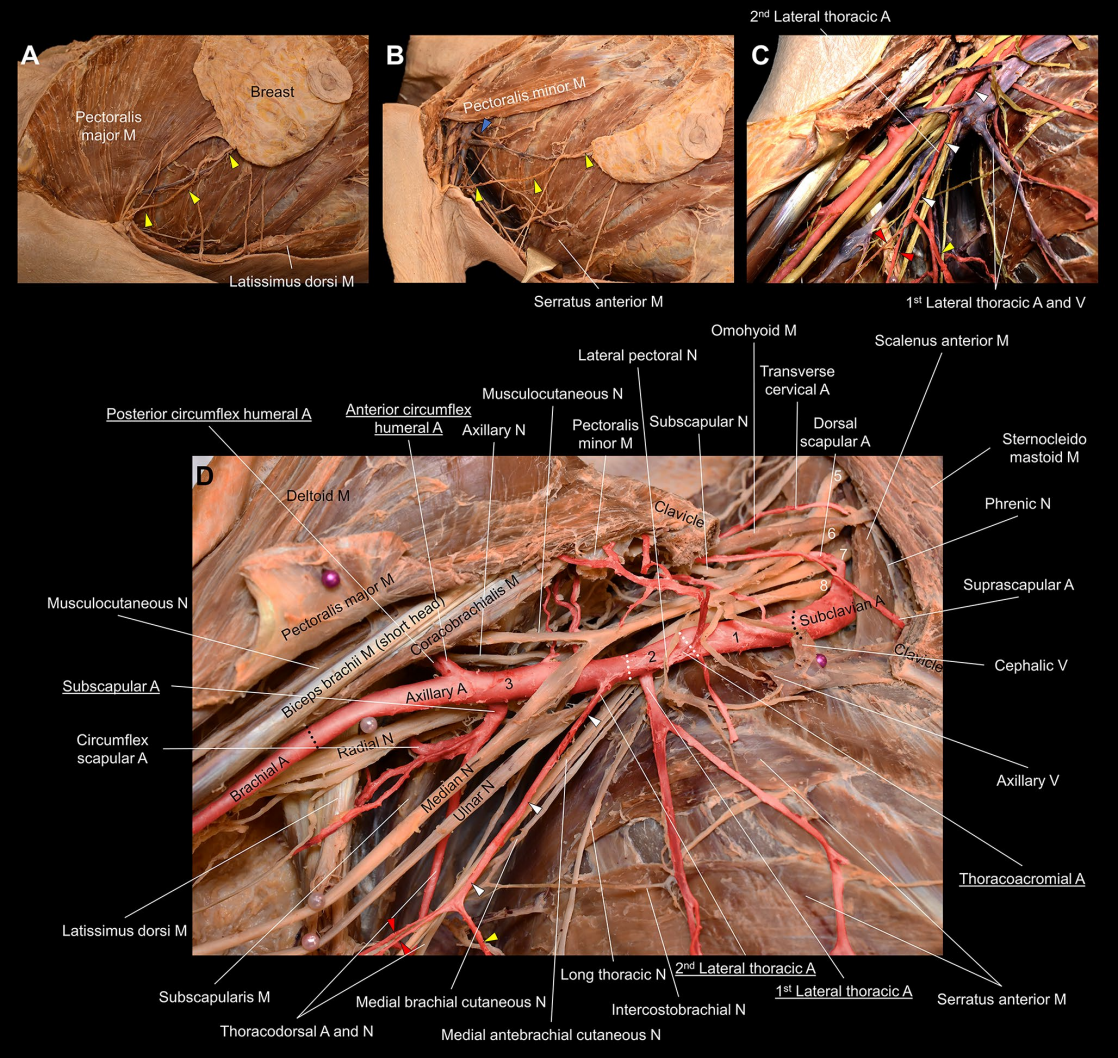
Progressive depiction of the right duplicate lateral thoracic arteries. Panel **A** depicts the emergence of the 2nd lateral thoracic artery (LTA) (yellow arrowheads) from the axilla, inferior to the pectoralis major, and its course directly into the breast tissue. Panel **B**, with the pectoralis major removed, exhibits the 1st LTA (blue arrowhead) emerging from the inferior margin of the pectoralis minor. The 2nd LTA originates from the lateral border of the axilla and courses far inferior to and parallel to the inferior margin of the pectoralis minor. After the removal of the pectoralis minor, Panels **C** and **D** depict the axillary anatomy, with (**C**) and without (**D**) the veins. Panel **C** highlights the course of the 2nd LTA, with the nerves digitally colored in yellow, veins in purple, and arteries in red. The 1st LTA originates from the 2nd part of the axillary artery, posterior to the pectoralis minor tendon, and has a typical course along the lateral thorax accompanied by the lateral thoracic vein. The 2nd LTA originates from the 3rd part of the axillary artery; the 1st portion (white arrowheads) takes an oblique course respective to the axillary artery. The 2nd LTA then gives off multiple branches, including one that courses directly to the breast tissue (yellow arrowheads) and two smaller branches (red arrowheads) that course into the subcutaneous region of the medial upper arm. Panel **D** highlights the origin of the duplicate LTAs with other major branches of the axillary artery (underlined), including the thoracoacromial and subscapular arteries. The black dotted lines denote the margin of the axillar artery. The white dotted lines divide the three parts (numbers in black) of the axillary artery. The numbers in white indicate the visible cervical nerve roots composing the brachial plexus. A, artery; M, muscle; N, nerve; V, vein

**Fig. 2:**
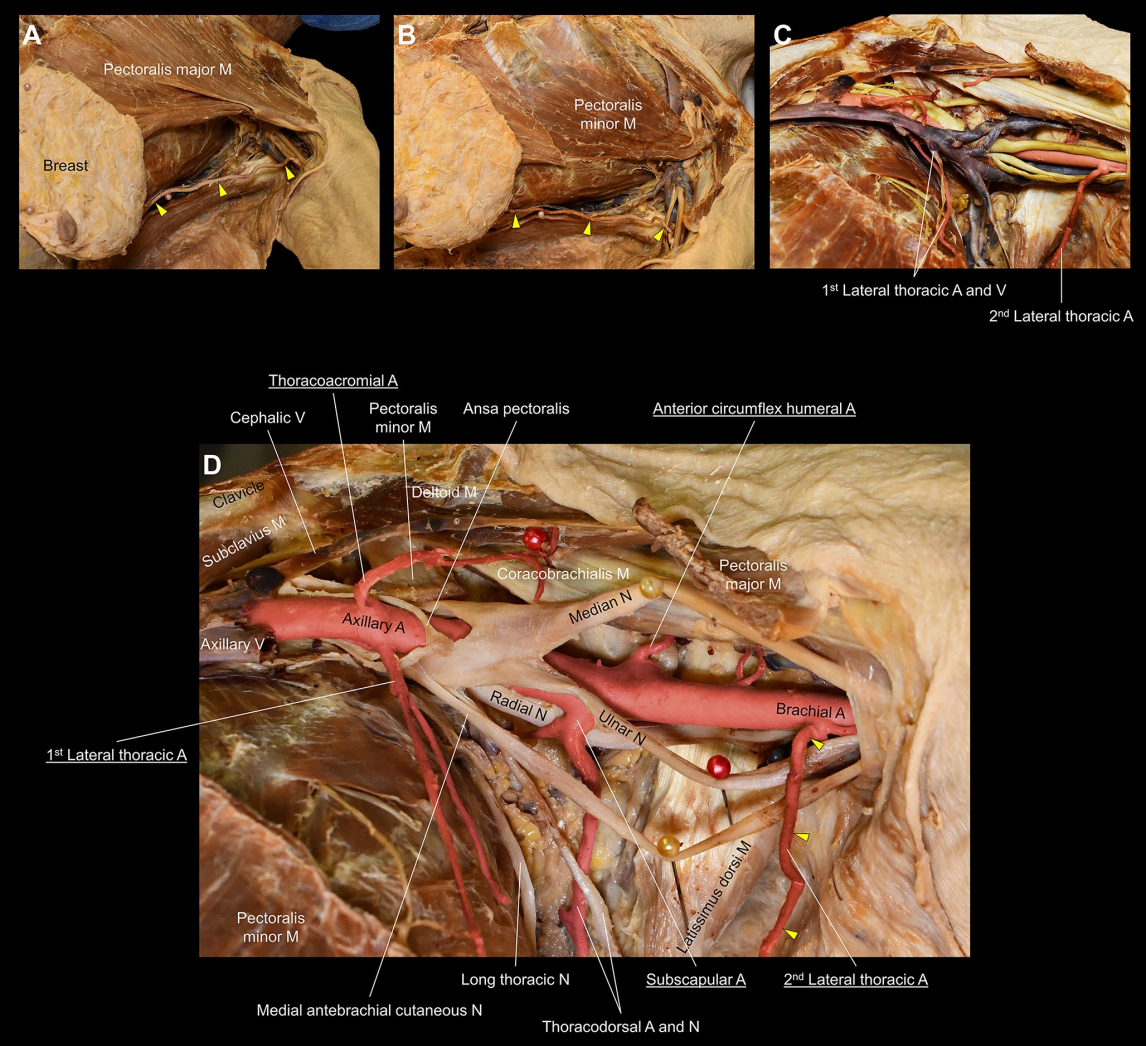
Progressive depiction of the left duplicate lateral thoracic arteries. Panels **A**, **B**, **C**, and **D** depict the progressive dissection of the left axilla. Panel **A** depicts the full course of the 2nd lateral thoracic artery (LTA) (yellow arrowheads), with an origin at the brachial artery, through the axilla and directly to the breast tissue. Panel **B** exhibits the same anatomy with the pectoralis major removed. Panel **C**, with the pectoralis minor removed, highlights the 1st LTA and lateral thoracic vein along with the 2nd LTA, with the nerves digitally colored in yellow, veins in purple, and arteries in red. Panel **D**, with the veins removed, highlights the origins of the duplicate LTAs with other major branches of the axillary artery (underlined), including the thoracoacromial and subscapular arteries. The 1st LTA originates from the 2nd part of the axillary artery, almost identical to the origin and course of the 1st LTA on the right. A, artery; M, muscle; N, nerve; V, vein

We received an 80-year-old female from the UCLA Donated Body Program. The donor was 5 feet and 4 inches tall and had a BMI of 15.4. The primary cause of death was attributed to Alzheimer’s Disease. During the standard anatomical dissection with photographic documentation of the thorax and both arms, an unreported duplicate variant of the LTA was discovered, bilaterally (Figs. 1 and 2).

On the right side (Fig. 1), the 1st LTA, with a classic retropectoral origin, appears to perfuse the chest wall and pectoralis minor. The 2nd LTA, termed the duplicate variant, with an origin from the 3rd part of the axillary artery, has a unique pathway and appears to play a much larger role for the breast tissue than the 1st LTA, as it courses directly into the breast tissue towards the nipple. The 2nd LTA, at first, courses toward the lateral wall of the axilla, almost parallel to the axillary sheath, then splits into one branch that courses back to the breast to converge with the LTV and two smaller branches that course to the skin of the medial upper arm, termed brachial branches. Accordingly, this 2nd LTA emerges from the axilla at the inferolateral margin of the pectoralis major and takes a more oblique course parallel to the inferior margin of the pectoralis minor to reach the breast.

On the left side (Fig. 2), the 1st LTA is symmetrical to the right 1st LTA, with a classic retropectoral origin and distribution along the lateral chest wall. The 2nd LTA also appears to play a major role in perfusion of the breast tissue, as it similarly courses directly into the breast tissue. However, unlike the right 2nd LTA, the left 2nd LTA is much more direct in its course through the axilla and has an origin from the brachial artery.

## Discussion

Traditionally, the LTA courses intimately with the LTV, running vertically along the lateral chest wall. However, in this case with bilaterally duplicated LTAs, the right 2nd LTA has a unique oblique path that courses along the axillary sheath to the lateral axilla and alters course to eventually come to a convergence with the LTV as it nears the breast tissue. This 2nd LTA, which also has small subcutaneous branches perfusing the medial upper arm, should be considered the main LTA due to its role in breast perfusion, as evident by its direct entry into the breast and course toward the nipple (Fig. 1). The left 2nd LTA uniquely originates from the brachial artery and similarly should be considered the main LTA due to its direct entry into the breast tissue (Fig. 2). Both LTA duplicate variants have not been reported in current literature [4–6]. The unique course of both variant LTAs increases the potential risk of inadvertent injury, stressing the importance of procedural imaging in the discovery of potential variations.

The results of hundreds of recorded dissections of the axillary region indicate significant variation in the origin of the LTA [4–6]. A textbook origin of the LTA from the 2nd part of the axillary artery does not always hold true. Loukas et al. found that the LTA arose most commonly from the thoracoacromial artery (67.62%), followed by the textbook origin of the 2nd part of the axillary artery (17.02%), the thoracodorsal artery (5%), and the subscapular artery (3.93%). Multiple/duplicate LTAs (3.09%) were also reported along with cases where no LTAs (3.33%) were present [5]. Olinger et al. found that the LTA arose most commonly from its textbook axillary artery origin (78.3%) along with an origin from the subscapular artery (4.2%). They also noted additional variation with a classic/textbook LTA that gives rise to the thoracodorsal (7.2%) and subscapular (5.4%) arteries [6]. Lee et al. recorded the LTAs originated from the 2nd part of the axillary artery (59.7%), 3rd part of the axillary artery (9.5%), thoracoacromial artery (2.6%), and the subscapular artery (21.6%) [4]. Importantly, however, the current form of duplicated variance with the right 2nd LTA’s convoluted course along with its brachial branches and the left 2nd LTA’s direct course from the brachial artery has never been reported bilaterally nor unilaterally (Figs. 1 and 2).

The embryological development of limb arterial supply is a process of formation, maturation, remodeling, and differentiation [7]. Variation in arteries is observable once differentiation, the process of primitive blood vessels, often capillaries, becoming enlarged, is achieved. This process is coupled with maturation of vessel walls, development of smooth muscle, and formation of the three tunic layers. The formation of more mature and larger vessels ensures that they can accommodate higher blood pressure and supply recipient tissues [1]. Ultimately, differentiation of certain vessels over others and specification of arterial fate is driven by vascular endothelial growth factor and Notch signaling, and shear stress-induction maintains arterial structure/vascular integrity after circulation is established [1, 8]. In summary, LTA variants have formed due to the complex nature of persistence and regression of blood vessels, influence of signaling molecules, genetics, and environmental factors during embryological development [1, 3, 8].

Knowledge of anatomical variants involving the LTA is necessary for relevant surgical procedures, such as breast surgeries, axillary lymph node dissections, sentinel lymph node biopsies, chest wall reconstructions, trauma surgeries of the chest, or surgeries of the axillary sheath and its contents. Breast surgery often involves dissection of the lateral thoracic region. An LTA found more laterally than typical could lead to unexpected bleeding if not anticipated or identified. In this case, both 2nd LTA variants originate more laterally and penetrate directly into the breast tissue and thus are certain to pose as problems if not properly anticipated. Therefore, meticulous dissection is required to avoid unnecessary complications. Likewise, in breast reconstruction surgeries, it is important to preserve arteries supplying the breast and chest wall for tissue survival and utilization for surgical flaps. A duplicate LTA has the potential to be a useful blood supply for surgical free flaps and equally has the potential to be injured. Similarly for chest wall reconstructions or trauma surgeries of the chest, a duplicate variant LTA, if uninjured, can be an asset to surgery of the chest wall and has the potential to improve surgical outcomes. Additionally, it is important for surgeons to be aware of potential LTA variants to avoid iatrogenic injury to the artery during axillary lymph node dissection or sentinel lymph node biopsy in the case of lymphadenopathy, breast cancer, or even skin cancers such as melanoma since there are numerous lymph nodes clustered around vessels in the axilla region. Lastly, in classical anatomical orientation, surgery of the axillary sheath would not involve risk to the LTA because the LTA typically courses perpendicular to the sheath and its contents, making it apparent and evident. However, in this outlined case, the proximal origin of the right 2nd LTA variant begins its course parallel to and intimately associated with the axillary sheath at the 3rd part of the axillary sheath and proximal brachial fascia (Fig. 1). Thus, any surgery involving the 3rd part of the axillary artery, axillary vein, or the cords of the brachial plexus would inherently be in proximity to this duplicate LTA, and it is prudent to not mistake the artery as one structure with the fascial sheath to avoid iatrogenic injury. In contrast, the left 2nd LTA, with a brachial artery origin, has a much more direct course through the axilla and to the breast. It is necessary for surgeons to anticipate this major artery coursing directly through the axilla as well as to be aware that the brachial artery can give rise to it, as outlined in this case (Fig. 2), especially since anterior incisions are common in surgeries involving the proximal arm. Preprocedural imaging and appropriate three-dimensional reconstruction, such as the computed tomography depicted in the Supplementary Figure, is useful to appreciate highly variable anatomy around the LTA.

## Conclusion

This case report demonstrates bilateral anatomical variation of the duplicated LTAs. Both 2nd LTAs appear to play an important role in the perfusion of the breast tissue. This case report may improve overall anatomical understanding of the arterial variations present within the axilla, which ultimately may be useful to surgeons.

## Electronic supplementary material

Below is the link to the electronic supplementary material.

Supplementary Figure 1: Typical Duplicate Lateral Thoracic Arteries Panels **A** and **B** depict uncommon but typical duplicate lateral thoracic arteries of a 66-year-old female, taken from a contrast-enhanced computed tomographic dataset. Both are retropectoral in origin, course parallel to each other, and emerge from the inferior margin of the pectoralis minor muscle (green arrowheads)

## Data Availability

All data supporting the findings of this study are available within this manuscript and its supplementary information.

## Abbreviations

LTA: Lateral thoracic artery
LTV: Lateral thoracic vein
