# Supplementary figures and images for: Rare bilateral anatomical variation of the lateral thoracic artery: duplicated arteries with unique origins and pathways
Source: Surg Radiol Anat. 2025 Feb 18;47(1):80. doi: 10.1007/s00276-025-03587-y (PMC11836202; doi:10.1007/s00276-025-03587-y)

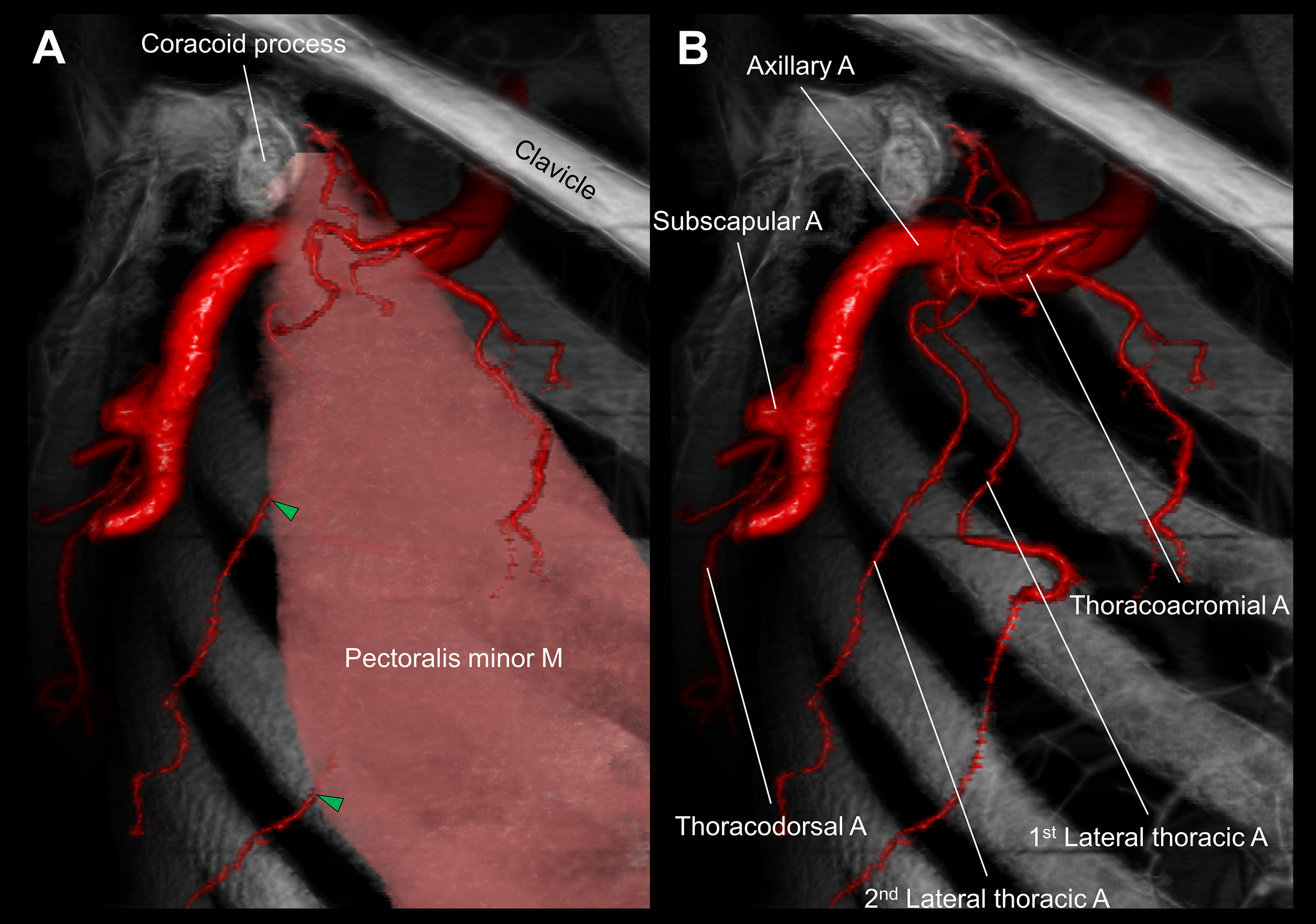

Supplement: Supplementary file 1 — Supplementary Figure 1: Typical Duplicate Lateral Thoracic Arteries Panels A and B depict uncommon but typical duplicate lateral thoracic arteries of a 66-year-old female, taken from a contrast-enhanced computed tomographic dataset. Both are retropectoral in origin, course parallel to each other, and emerge from the inferior margin of the pectoralis minor muscle (green arrowheads) [file 276_2025_3587_MOESM1_ESM.tif]
